# Supplementary material for: Decidual natural killer cells dysfunction is caused by IDO downregulation in dMDSCs with Toxoplasma gondii infection
Source: Commun Biol. 2024 May 31;7:669. doi: 10.1038/s42003-024-06365-5 (PMC11143278; doi:10.1038/s42003-024-06365-5)

Supplementary Fig. 1 Gating strategy for flow cytometry analysis.

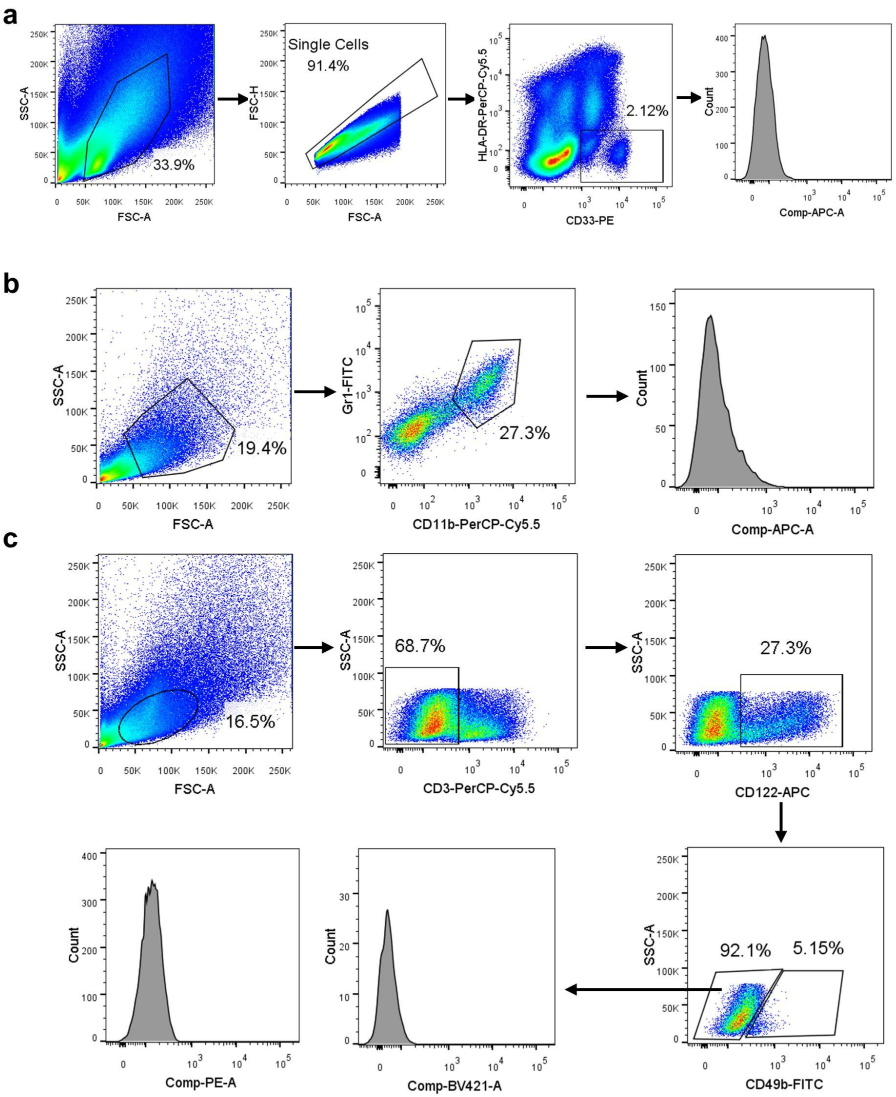

a Gating strategy of human dMDSCs. After single cells were gated by FSC-A and FSC-H, CD33+ HLA-DR − cells were gated as dMDSCs for further analysis. **b** Gating strategy of mouse dMDSCs. After cells were gated by FSC-A and SSC-A, CD11b+ Gr-1+ cells were gated as dMDSCs for further analysis. **c** Gating strategy of mouse dNK cells. After lymphocyte cells were gated by FSC-A and SSC-A, CD3 − CD122+CD49b − cells were gated as dNK cells for further analysis.

Supplementary Fig. 2 Uncropped blots images for western blot.

a. Uncropped blots of Figure 2b

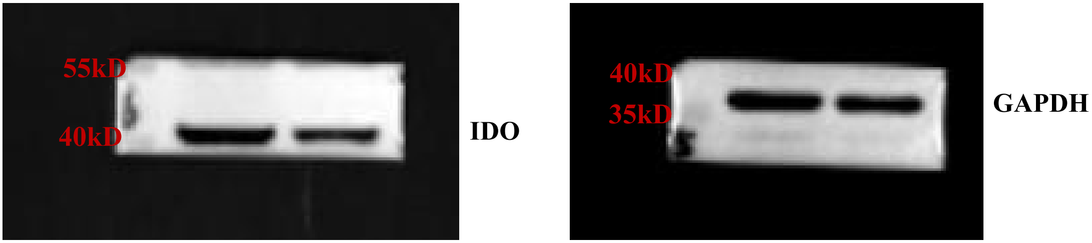

b. Uncropped blots of Figure 3b

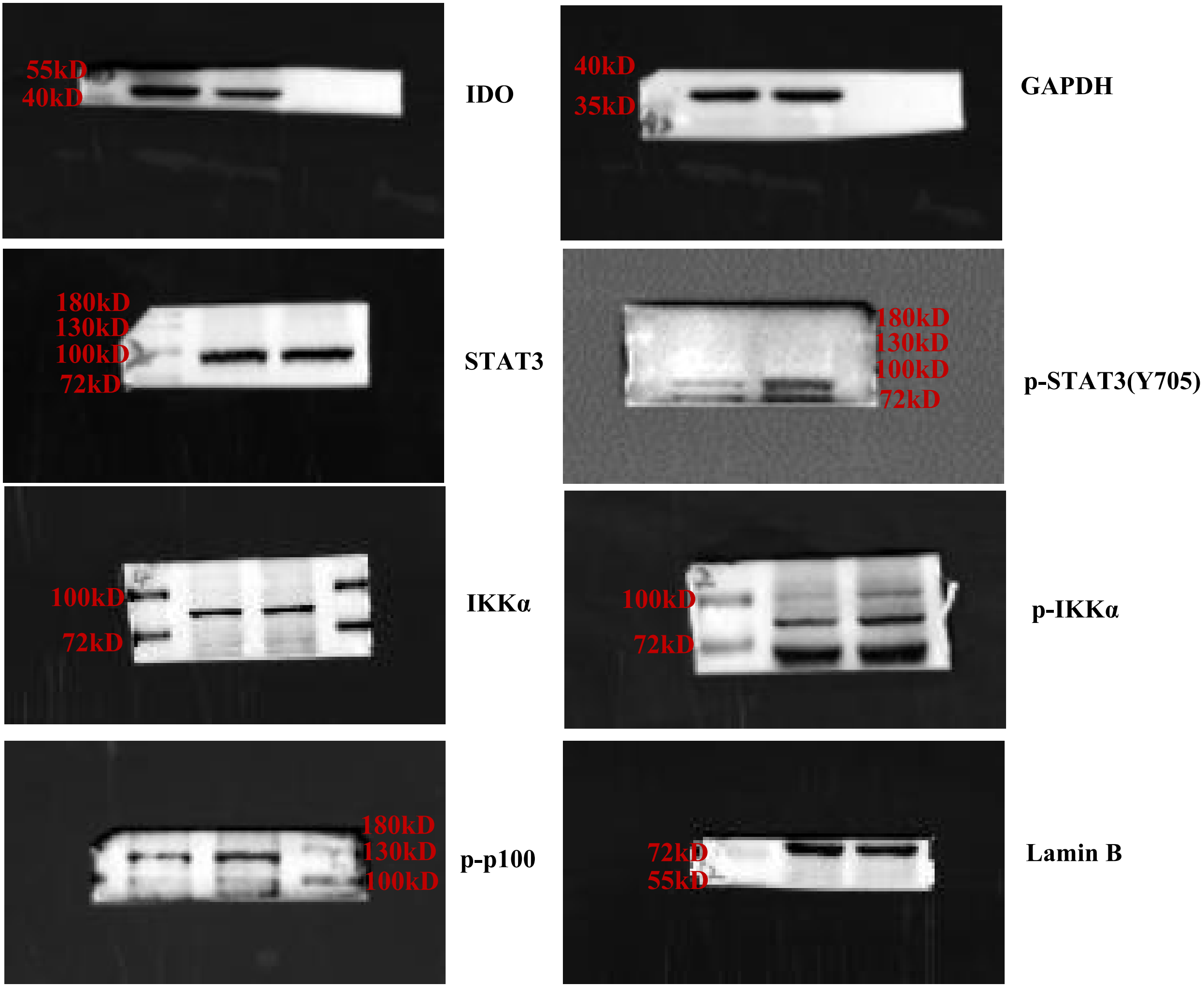

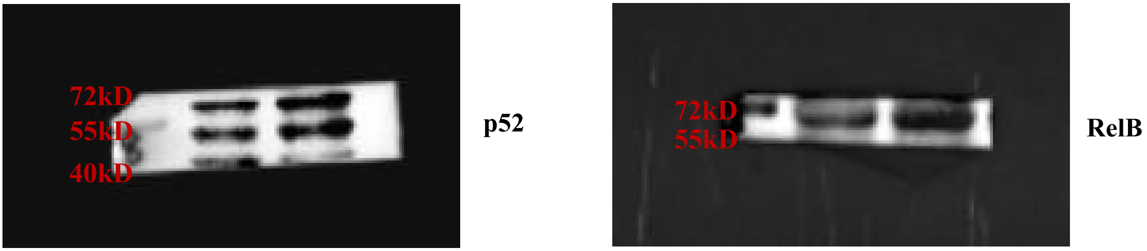

c. Uncropped blots of Figure 4b

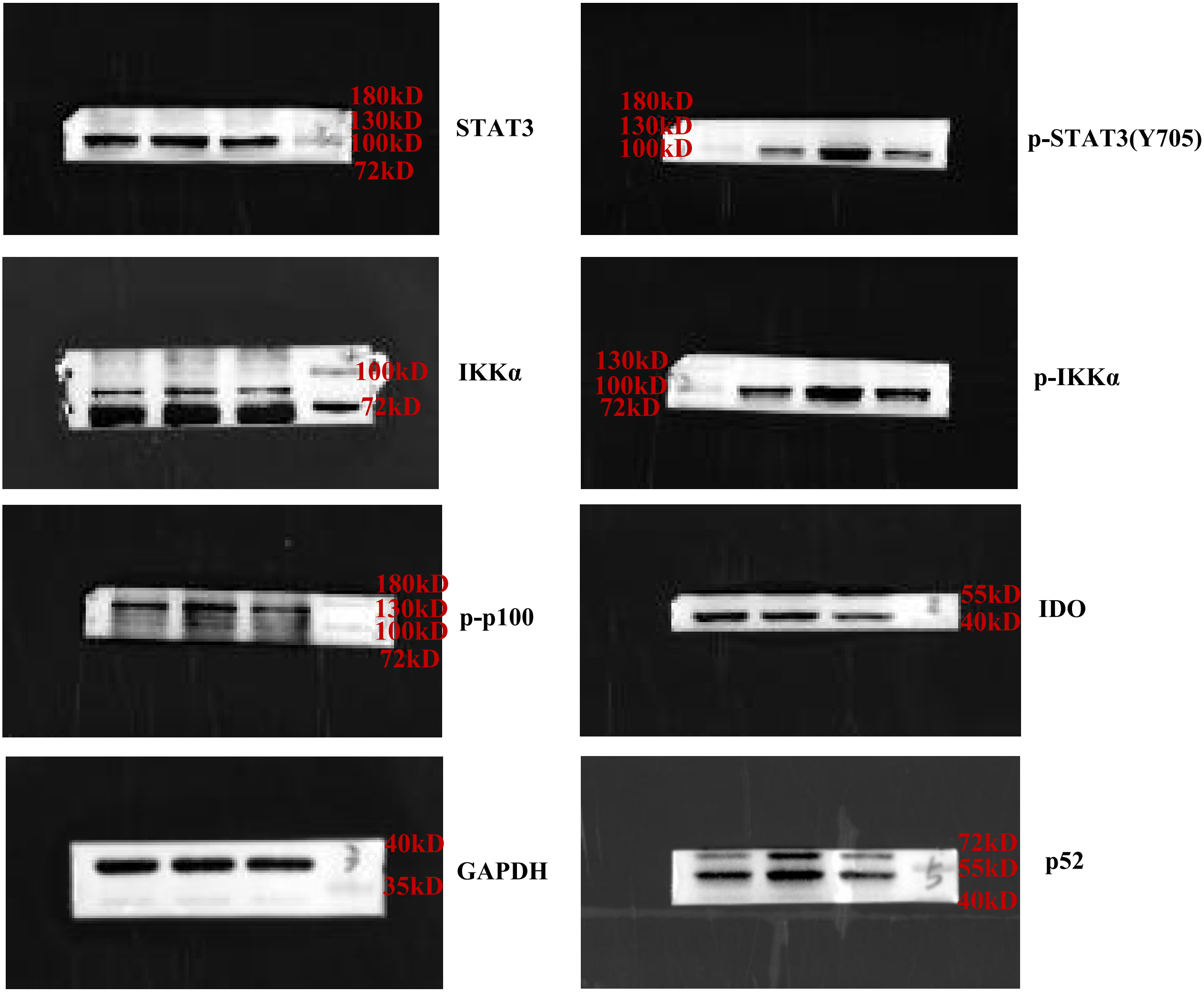

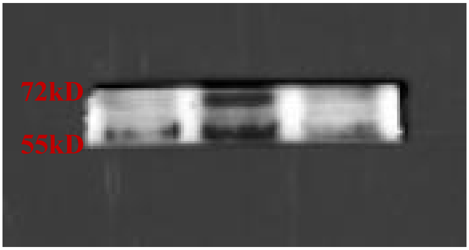

RelB

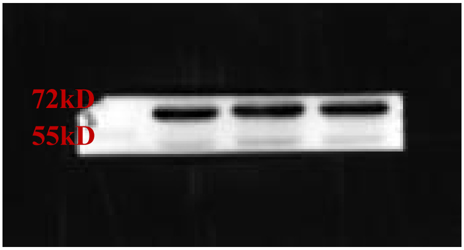

Lamin B

d. Uncropped blots of Figure 4c

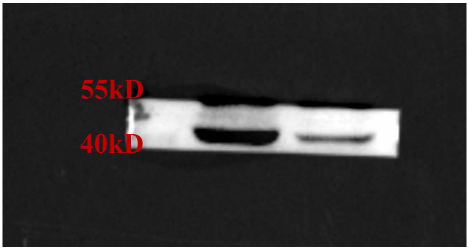

IDO

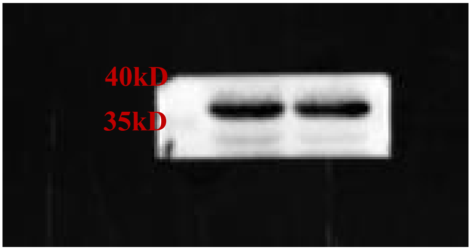

GAPDH

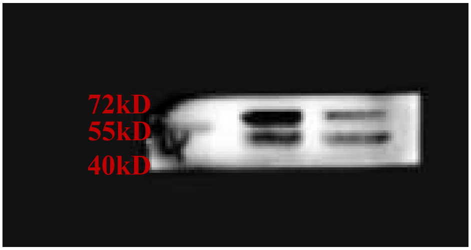

p52

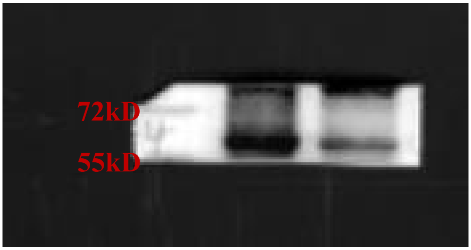

RelB

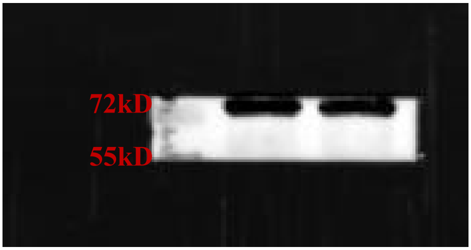

Lamin B

e. Uncropped blots of Figure 5b

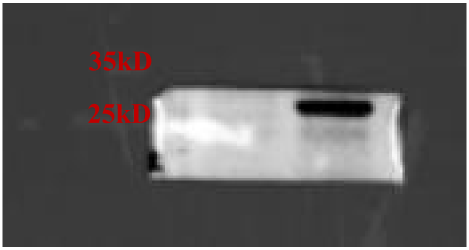

SOCS3

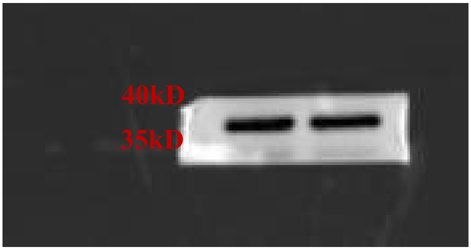

GAPDH

f. Uncropped blots of Figure 5c

WCL

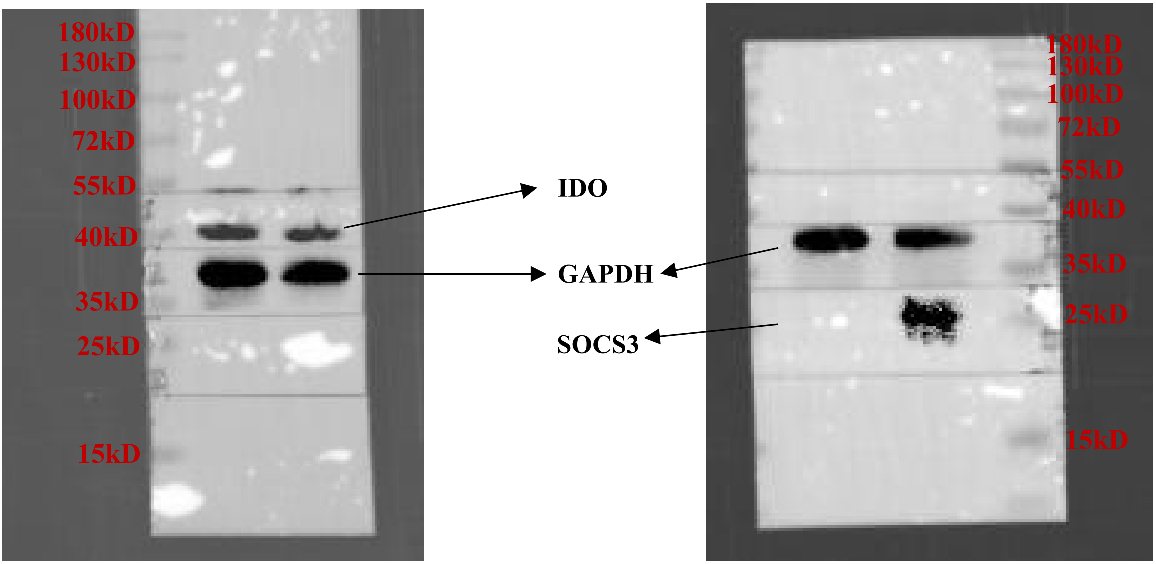

IP

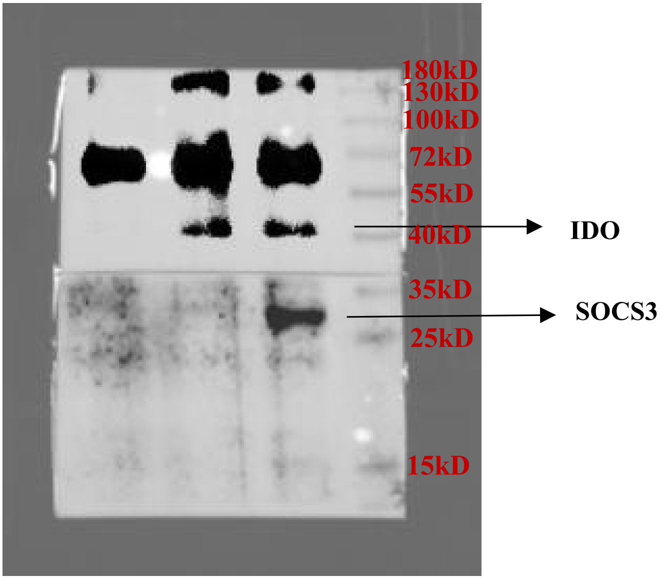

g. Uncropped blots of Figure 6c

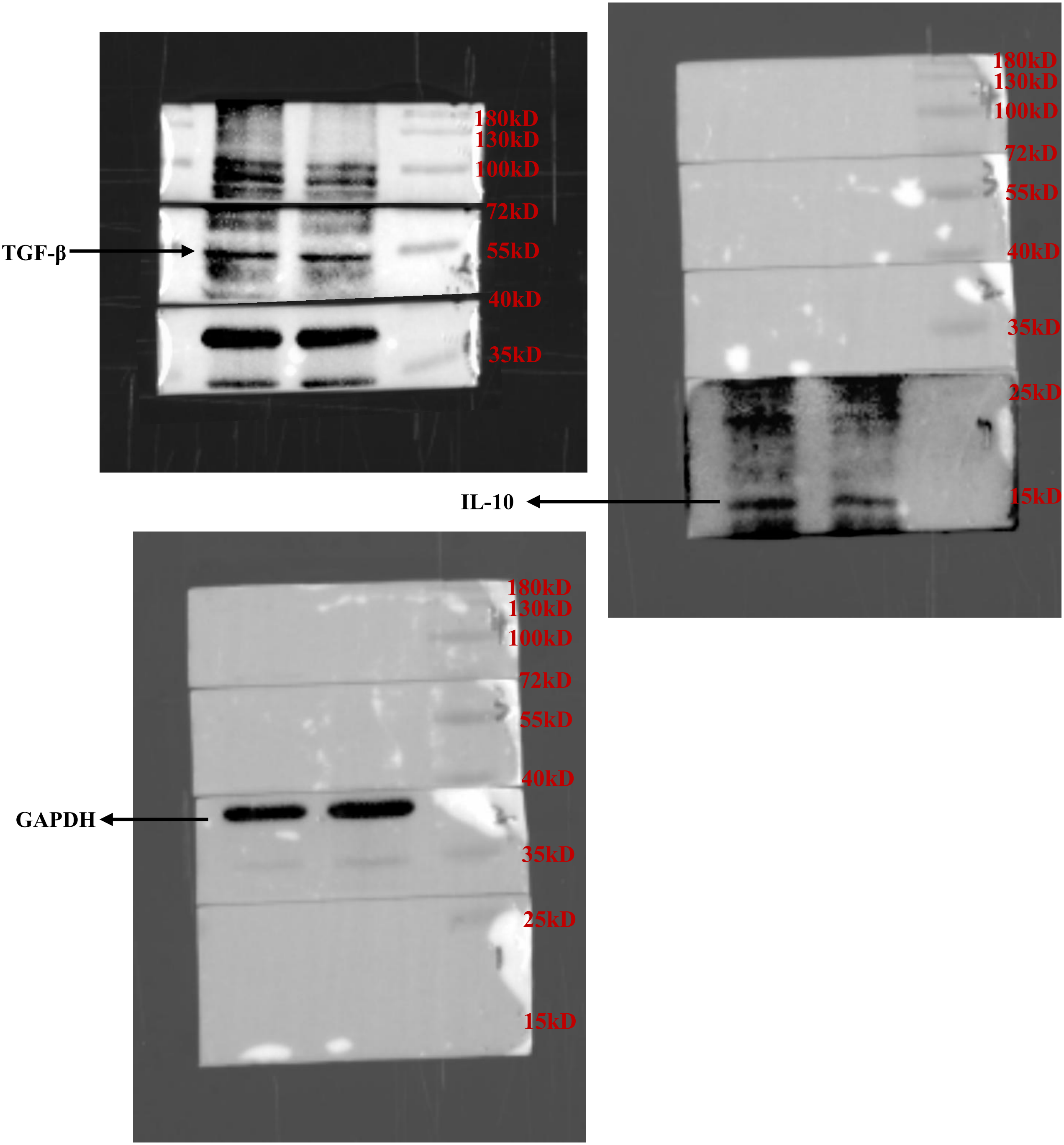

h. Uncropped blots of Figure 6e

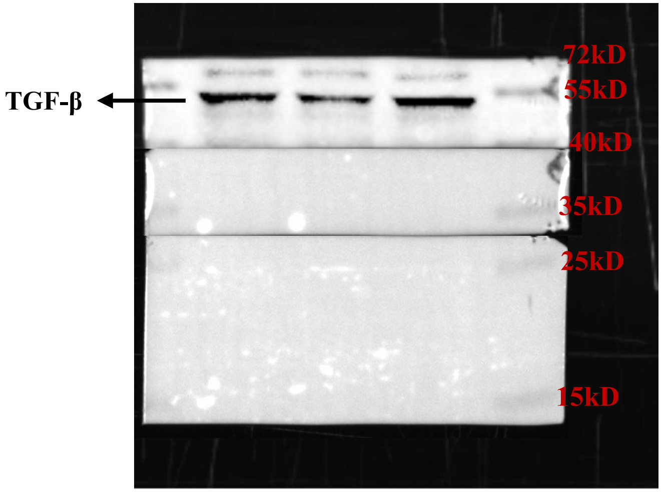

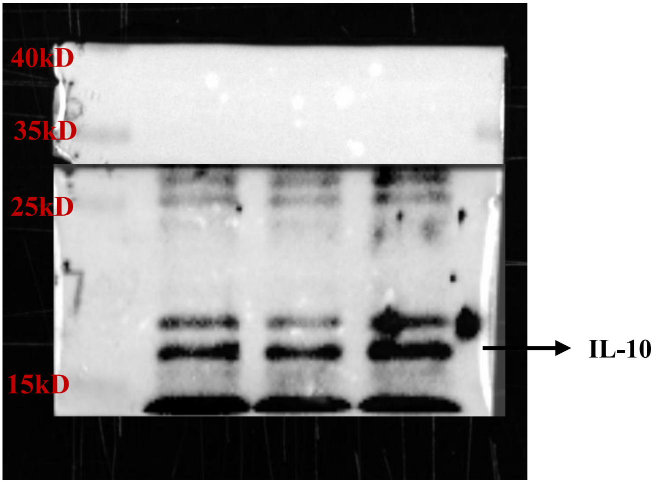

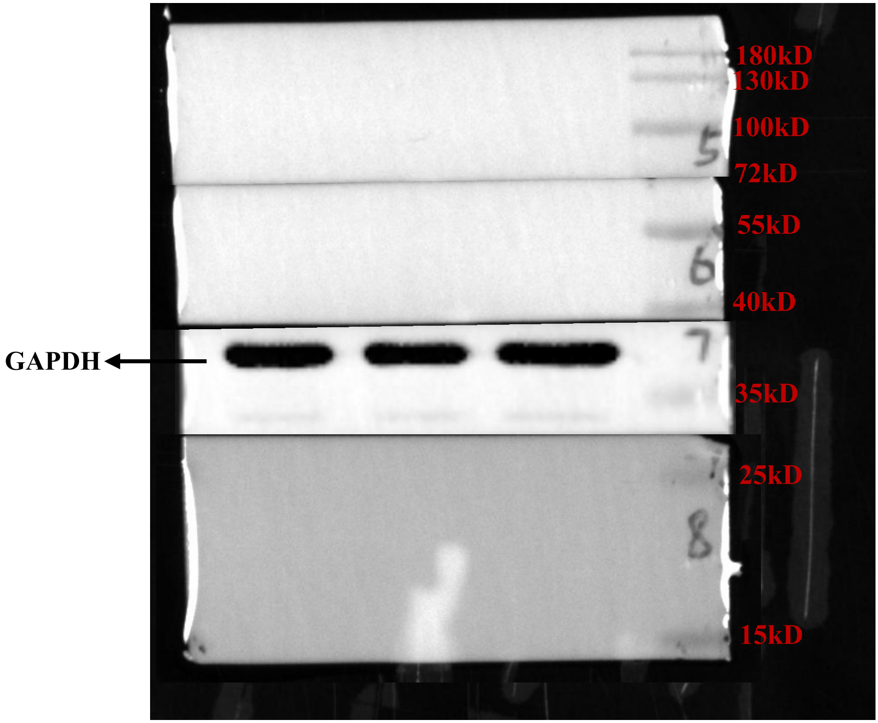

i. Uncropped blots of Figure 7a

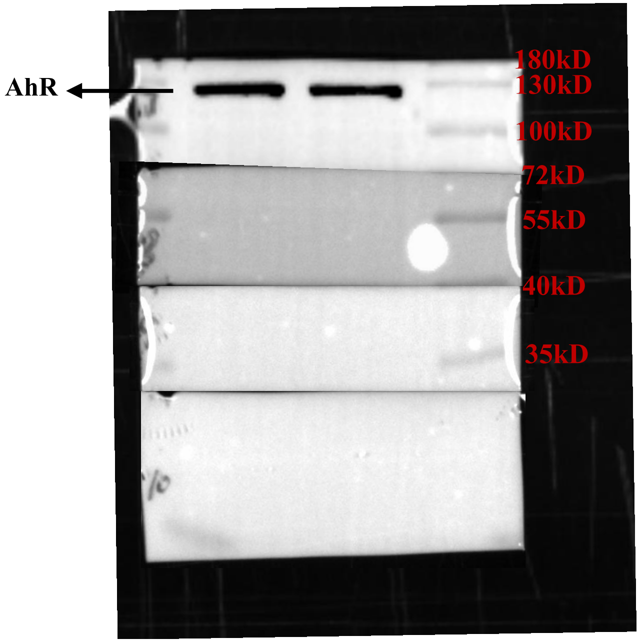

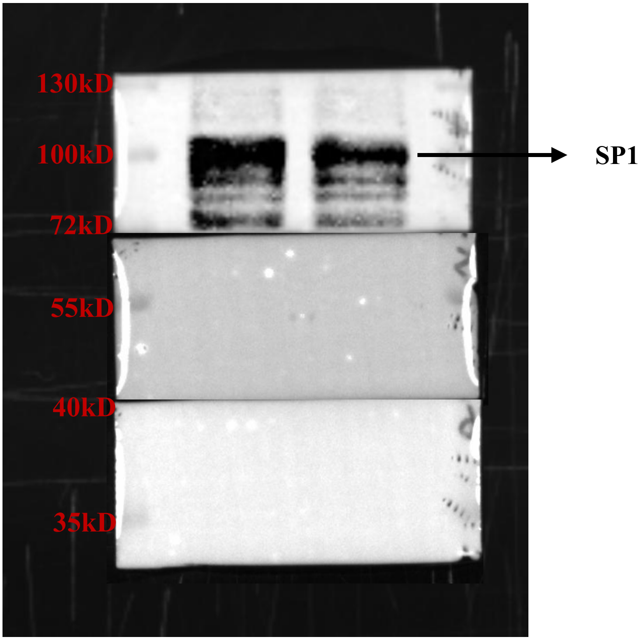

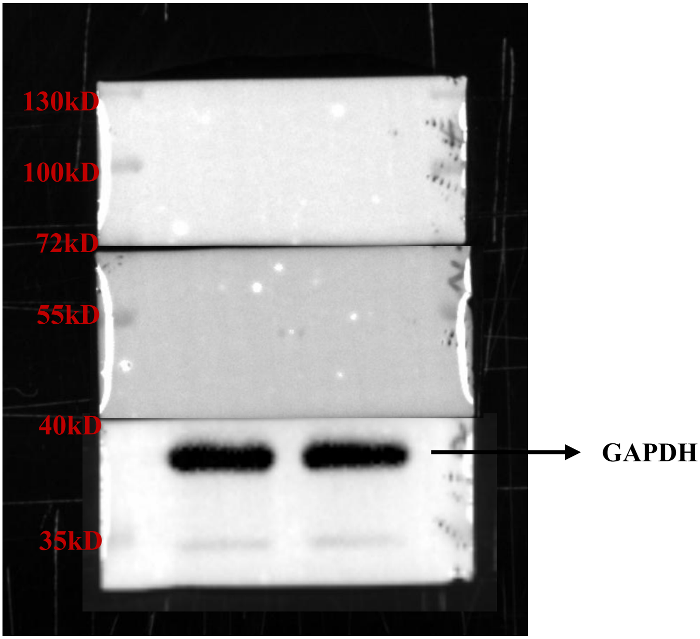

j. Uncropped blots of Figure 7b

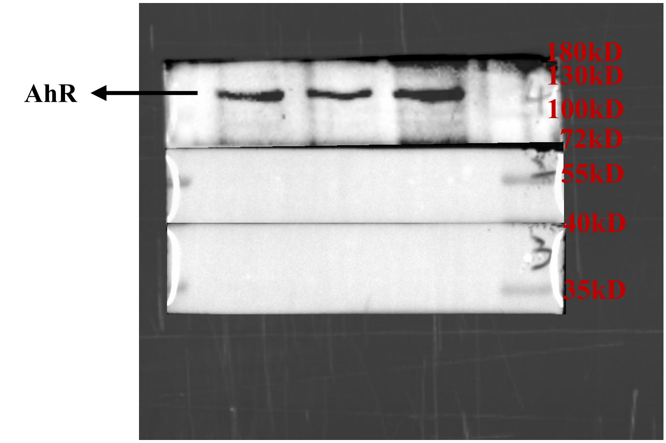

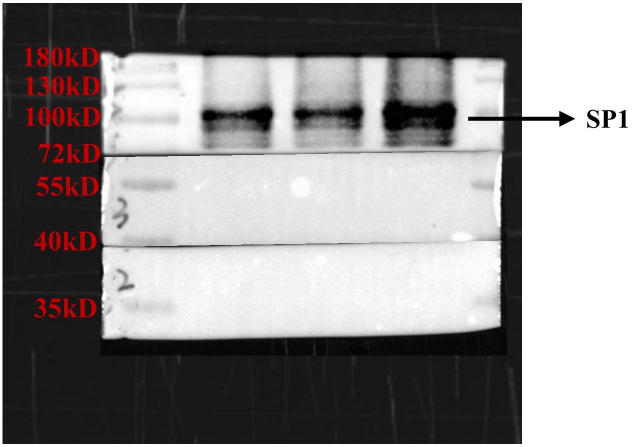

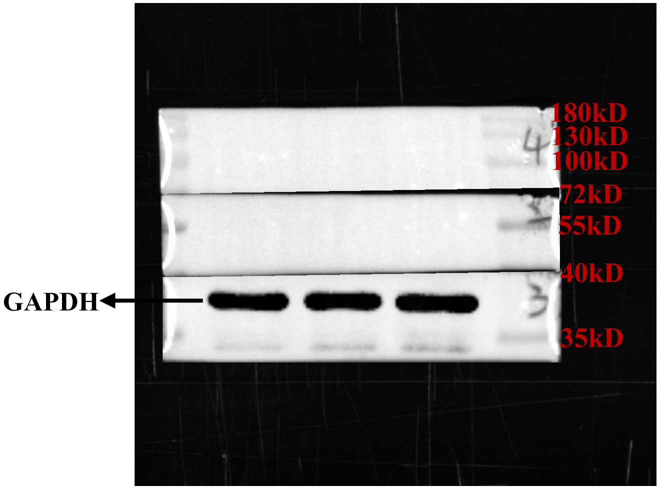

k. Uncropped blots of Figure 7e

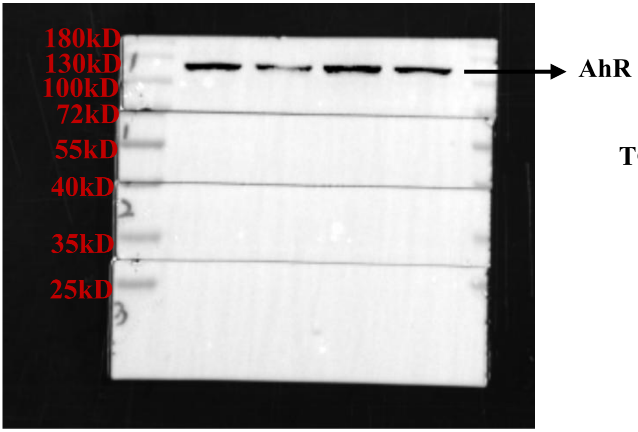

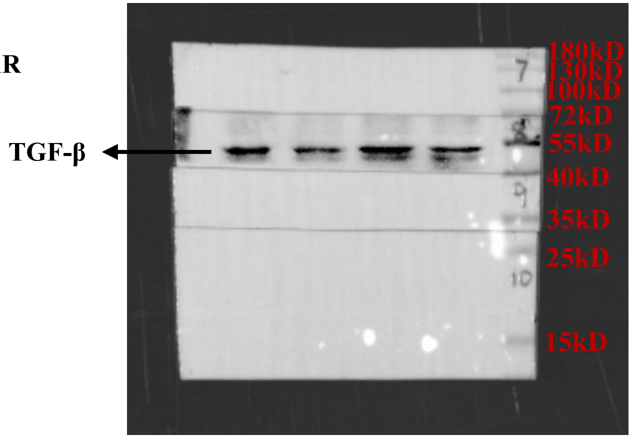

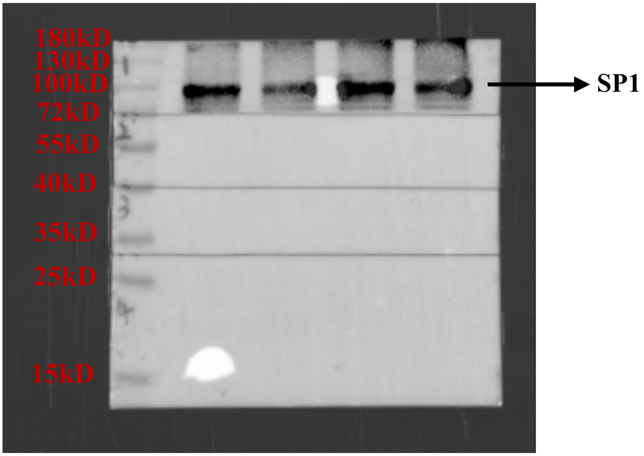

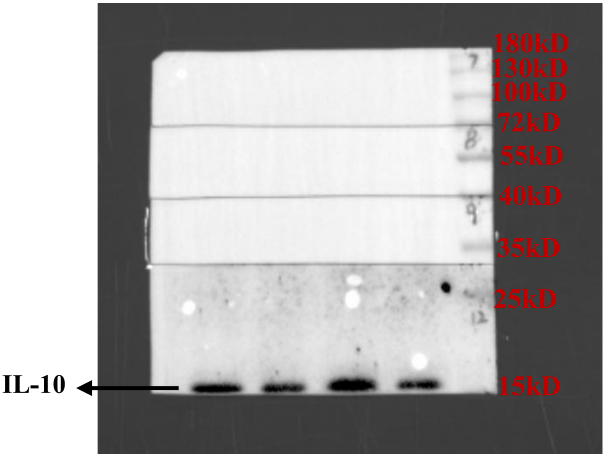

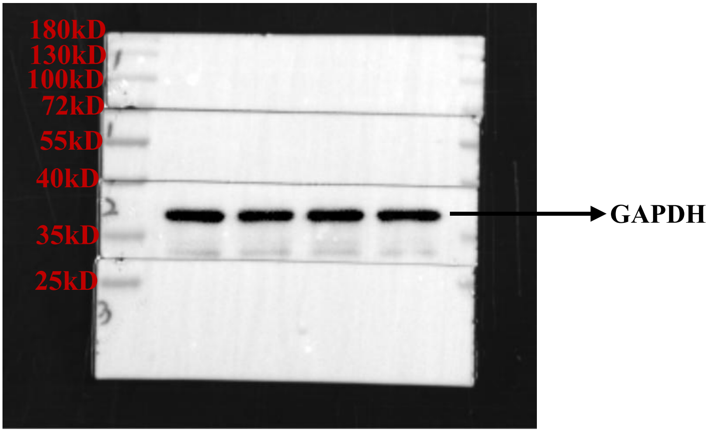

l. Uncropped blots of Figure 7f

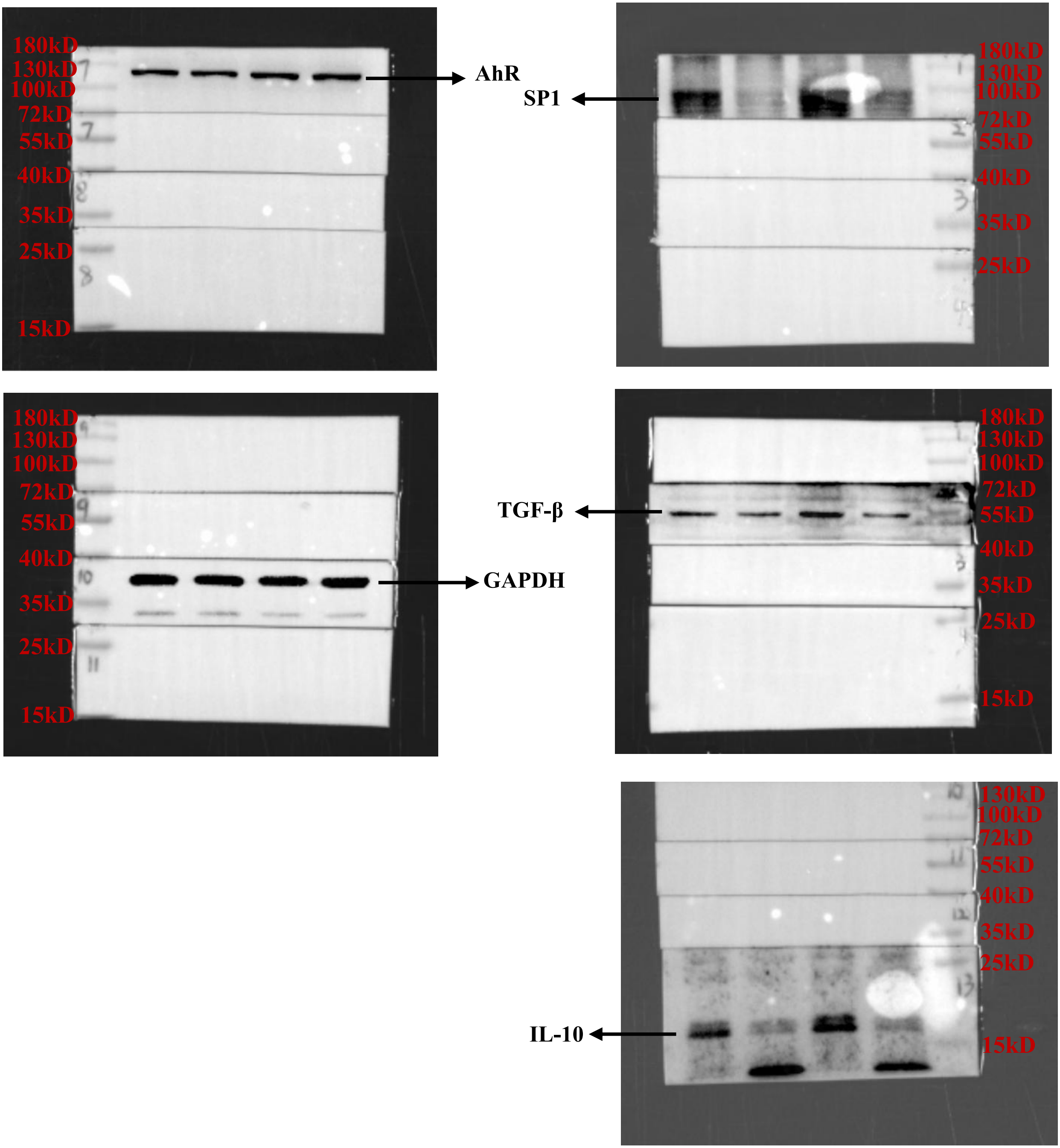

Supplement: Supplementary file 2 — Supplementary Information [file 42003_2024_6365_MOESM2_ESM.pdf]
