# Supplementary material for: Decidual natural killer cells dysfunction is caused by IDO downregulation in dMDSCs with Toxoplasma gondii infection
Source: Commun Biol. 2024 May 31;7:669. doi: 10.1038/s42003-024-06365-5 (PMC11143278; doi:10.1038/s42003-024-06365-5)
Supplement: Supplementary file 5 — Reporting Summary [file 42003_2024_6365_MOESM5_ESM.pdf]

nature portfolio

Corresponding author(s): Xuemei Hu

Last updated by author(s): May 18, 2024

Reporting Summary

Nature Portfolio wishes to improve the reproducibility of the work that we publish. This form provides structure for consistency and transparency in reporting. For further information on Nature Portfolio policies, see our Editorial Policies and the Editorial Policy Checklist.

Statistics

For all statistical analyses, confirm that the following items are present in the figure legend, table legend, main text, or Methods section.

n/a	Confirmed	
☐	☒	The exact sample size (*n*) for each experimental group/condition, given as a discrete number and unit of measurement
☐	☒	A statement on whether measurements were taken from distinct samples or whether the same sample was measured repeatedly
☐	☒	The statistical test(s) used AND whether they are one- or two-sided<br>*Only common tests should be described solely by name; describe more complex techniques in the Methods section.*
☐	☒	A description of all covariates tested
☐	☒	A description of any assumptions or corrections, such as tests of normality and adjustment for multiple comparisons
☐	☒	A full description of the statistical parameters including central tendency (e.g. means) or other basic estimates (e.g. regression coefficient) AND variation (e.g. standard deviation) or associated estimates of uncertainty (e.g. confidence intervals)
☒	☐	For null hypothesis testing, the test statistic (e.g. *F*, *t*, *r*) with confidence intervals, effect sizes, degrees of freedom and *P* value noted<br>*Give P values as exact values whenever suitable.*
☒	☐	For Bayesian analysis, information on the choice of priors and Markov chain Monte Carlo settings
☒	☐	For hierarchical and complex designs, identification of the appropriate level for tests and full reporting of outcomes
☒	☐	Estimates of effect sizes (e.g. Cohen's *d*, Pearson's *r*), indicating how they were calculated

Our web collection on statistics for biologists contains articles on many of the points above.

Software and code

Policy information about availability of computer code

Data collection	A FACSCanto II flow cytometer (BD Bioscience) was used for all flow cytometry data acquisition. A Bio-Rad iQ5 multicolor RT-PCR system was used for all quantitative real-time PCR (qPCR) data acquisition. A Bio-Red ChemiDoc XRS+ systemwas used for all western blot data acquisition.
Data analysis	Flow cytometry results were analyzed with FlowJo 10.0. Western blot results were analyzed with ImageJ. The statistical analyses were performed using the GraphPad Prism 9 Statistics software package.

For manuscripts utilizing custom algorithms or software that are central to the research but not yet described in published literature, software must be made available to editors and reviewers. We strongly encourage code deposition in a community repository (e.g. GitHub). See the Nature Portfolio guidelines for submitting code & software for further information.

Data

Policy information about availability of data

All manuscripts must include a data availability statement. This statement should provide the following information, where applicable:

- Accession codes, unique identifiers, or web links for publicly available datasets
- A description of any restrictions on data availability
- For clinical datasets or third party data, please ensure that the statement adheres to our policy

All data reported in this manuscript will be freely available. The source data can be found in Supplementary Data 1 and all uncropped blots can be found in Supplementary Fig. 2. The datasets are available form the corresponding author on reasonable request.

Research involving human participants, their data, or biological material

Policy information about studies with human participants or human data. See also policy information about sex, gender (identity/presentation), and sexual orientation and race, ethnicity and racism.

Reporting on sex and gender	Human specimens was all collected form female decidual tissue, because this present study is to research the effects of Toxoplasma. gondii on the maternal-fetal interface in early pregnancy.
Reporting on race, ethnicity, or other socially relevant groupings	No race, ethnicity, or other socially relevant groupings were included in the study.
Population characteristics	The human decidual tissues of early pregnancy were collected from healthy pregnant women of 6–8 weeks of gestational age who had voluntarily aborted.
Recruitment	The samples were collected from the abortion clinic of Yantai Affiliated Hospital, Binzhou Medical University, the Yantai Zhifu District Material and Child Health Hospital.
Ethics oversight	The sample collection procedure for this study was approved by the Ethics Committee of Binzhou Medical University (approval number 2017-016-01).

Note that full information on the approval of the study protocol must also be provided in the manuscript.

Field-specific reporting

Please select the one below that is the best fit for your research. If you are not sure, read the appropriate sections before making your selection.

☒ Life sciences ☐ Behavioural & social sciences ☐ Ecological, evolutionary & environmental sciences

For a reference copy of the document with all sections, see nature.com/documents/nr-reporting-summary-flat.pdf

Life sciences study design

All studies must disclose on these points even when the disclosure is negative.

Sample size	Sample size was determined based on the similar study in this field, and was chosen to ensure that the statistical power were satisfied and that experimental effects were found.
Data exclusions	No data were excluded.
Replication	Reported experiments were repeated at least 3 times.
Randomization	Human samples and animals were randomly assigned for the different group in this study.
Blinding	The researcher did not blind to the experimental groups. The analysis of the conclusions was based on the quantitative parameters and the statistical significance of objective data, which is independent of observer subjectivity and psychological expectation.

Reporting for specific materials, systems and methods

We require information from authors about some types of materials, experimental systems and methods used in many studies. Here, indicate whether each material, system or method listed is relevant to your study. If you are not sure if a list item applies to your research, read the appropriate section before selecting a response.

Materials & experimental systems

n/a	Involved in the study	
☐	☒	Antibodies
☒	☐	Eukaryotic cell lines
☒	☐	Palaeontology and archaeology
☐	☒	Animals and other organisms
☒	☐	Clinical data
☒	☐	Dual use research of concern
☒	☐	Plants

Methods

n/a	Involved in the study	
☒	☐	ChIP-seq
☐	☒	Flow cytometry
☒	☐	MRI-based neuroimaging

Antibodies

Antibodies used

The western blot antibodies in this study include: STAT3 (Proteintech, China, 10253-2-AP, 1:2000), p-STAT3 (CST, UK, 9145S, 1:1000), IDO (Abcam, UK, ab76157, 1:1000), IKKα (Wanleibio, China, WL00053, 1:500), p- IKKα (ABclonal, China, AP0506, 1:1000), p-p100 (ABclonal, China, AP1367, 1:500), p52 (Proteintech, China, 15503-1-AP, 1:500), RelB (Proteintech, China, 66947-1-Ig, 1:2000), SOCS3 (Wanleibio, China, WL01364, 1:1000), GAPDH (Proteintech, China, 10494-1-AP, 1:40000), LaminB (Wanleibio, China, WL01775, 1:1000), AhR (Wanleibio, China, WL02657, 1:3000), SP1 (Proteintech, China, 21962-1-AP, 1:3000), TGF-β (Bioss, China, bs-0086R, 1:1000), IL-10 (Wanleibio, China, WL03088, 1:1000) and IDO (Abcam, UK, ab211017, 1:1000, IP).The flow cytometry antibodies in this study include: PERCP-CY5.5-CD11b (Biolegend, USA, 101228), FITC-Gr1 (Biolegend, USA, 108406), eFluor 660-IDO (Invitrogen, USA, 50-9473-82), APC- p-STAT3 (Tyr705) (Invitrogen, USA, 17-9033-41), PERCP-CY5.5-CD3e (Invitrogen, USA, 45-0031-82), APC-CD122 (Invitrogen, USA,17-1222-82), FITC-CD49b (Biolegend, USA, 108906), PE-AhR (Invitrogen, USA, 12-5925-82) and BV421-TGF-β (Biolegend, USA, 141408), PE-CD33 (Biolegend, USA, 303404), FITC-HLA-DR (Invitrogen, USA, 11-9956-42), APC-IDO (Invitrogen, USA, 17-9477-42), and BV421-p-STAT3 (Tyr705) (Biolegend, USA, 651009).

Validation

All antibodies employed in this study are sourced from the specified commercial providers.

Animals and other research organisms

Policy information about studies involving animals; ARRIVE guidelines recommended for reporting animal research, and Sex and Gender in Research

Laboratory animals

Wild-type C57BL/6 mice (6–8-week-old females and 8–10-week-old males) were purchased from the Pengyue Laboratory Animal Technology Co., Ltd. (Jinan, China). The IDO-deficient mice were successfully bred by the Nanjing Model Animal Center (Nanjing, China) with the background of C57BL/6.

Wild animals

This study did not involve wild-animals.

Reporting on sex

The mice used in the experimental group were all female.

Field-collected samples

This study did not utilize samples collected form the field.

Ethics oversight

Animals' experiments were conducted according to the ethical standards of the Ethics Committee and Institutional Animal Experimental Ethics Committee of Binzhou Medical University (permit number 2017-009-09).

Note that full information on the approval of the study protocol must also be provided in the manuscript.

Plants

Seed stocks

This study not involve plants.

Novel plant genotypes

This study not involve plants.

Authentication

This study not involve plants.

Flow Cytometry

Plots

Confirm that:

☒ The axis labels state the marker and fluorochrome used (e.g. CD4-FITC).

☒ The axis scales are clearly visible. Include numbers along axes only for bottom left plot of group (a 'group' is an analysis of identical markers).

☒ All plots are contour plots with outliers or pseudocolor plots.

☒ A numerical value for number of cells or percentage (with statistics) is provided.

Methodology

Sample preparation

For mouse:On Gd 13, the uterus and placental tissue were carefully separated and washed three times with cold PBS. The tissues were cut into 1–3 mm fragments using ophthalmic scissors. Then, the fragments were suspended in RPMI 1640 medium, 1 mg/ml of collagenase IV (Biofroxx, Germany) and 0.2 mg/ml of DNase I (Sigma-Aldrich, St. Louis, USA) were added. The samples were digested in a biochemical incubator at 37°C for 40 min. The digested tissues were filtered through 48 µm

sterile nets and collected in 50 ml centrifuge tubes. The cells were removed by centrifugation at 700 × g for 10 min, the supernatant was discarded, and the precipitate was resuspended in cold PBS. The mononuclear cells were collected from a white membrane layer in the mouse lymphocyte isolation medium (TBD Science, China) after Ficoll density gradient centrifugation. Finally, the cells were collected, resuspended in cold PBS, and used for subsequent flow cytometry analysis.
For human: The decidual tissues were washed 4–5 times with cold PBS to remove the blood and then cut into small fragments measuring 1–3 mm using ophthalmic scissors. Then, the fragments were suspended in RPMI 1640 medium,1 mg/ml of collagenase IV (Biofroxx, Germany) and 0.2 mg/ml of DNase I (Sigma-Aldrich, St. Louis, USA) were added to digest the tissues in a biochemical incubator at 37°C for 50–60 min. The digested tissues were filtered through 48 μm sterile nets and collected in 50 ml centrifuge tubes. The cells were removed by centrifugation at 700 × g for 10 min, the supernatant was discarded, and the precipitate was resuspended in cold PBS. The mononuclear cells were collected from the white membrane layer using human lymphocyte isolation medium (TBD Science, China) after Ficoll density gradient centrifugation. Finally, the cells were collected, resuspended in cold PBS, and used for subsequent flow cytometry analysis.
The prepared human and mice decidual mononuclear cell suspensions were stained with surface markers. After surface marker staining, the cells were fixed and permeabilized using CytoFix/Perm solution (Foxp3/Transcription Factor Staining Buffer Set, eBioscience) and stained with p-STAT3, IDO, AhR and TGF-β antibodies following the manufacturer’s instructions.

Instrument

FACSCanto II flow cytometer (BD Bioscience)

Software

FlowJo 10.0

Cell population abundance

Details are in the "Methods" section.

Gating strategy

All gating strategy are in Supplementary Figure 1.

☒ Tick this box to confirm that a figure exemplifying the gating strategy is provided in the Supplementary Information.
